# Is Marfan Syndrome Associated with Primary Structural Changes in the Left Atrium?

**DOI:** 10.3390/diagnostics13203278

**Published:** 2023-10-23

**Authors:** Kun Zhang, Lucas Ernst, Isabel Schobert, Karla Philipp, Georg Böning, Frank R. Heinzel, Leif-Hendrik Boldt, Petra Gehle

**Affiliations:** 1 Department of Internal Medicine and Cardiology, Charité—Universitätsmedizin Berlin, Corporate Member of Freie Universität Berlin, Humboldt Universität zu Berlin, and Berlin Institute of Health, 10117 Berlin, Germany; 2 German Centre for Cardiovascular Research (DZHK), 10785 Berlin, Germany; 3 Department of Radiology, Charité—Universitätsmedizin Berlin, Corporate Member of Freie Universität Berlin, Humboldt Universität zu Berlin, and Berlin Institute of Health, 10117 Berlin, Germany; 4 2. Medical Clinic, Municipal Clinic Dresden, 01324 Dresden, Germany

**Keywords:** Marfan syndrome, cardiac function, echocardiography, aorta, cardiomyopathy, left atrium, biomarkers, NT-proBNP, aorto-ventricular coupling

## Abstract

Marfan syndrome (MFS) is an autosomal-dominant multisystem connective tissue disorder that is based on mutations in the *FBN1* gene and variably affects different organs, including the heart. In this study, we investigated cardiac function with a focus on the left atrium (LA) in a relatively large cohort of patients with MFS. After screening of 1165 patients that had been examined in our center between 2016 and 2020, 231 adult MFS patients with and without aortic operation were included in our study and compared to a healthy control group (*n* = 106). Cardiac function was assessed by transthoracic echocardiography and NT-proBNP was used as a secretory marker. Most (94.8%) of the patients received genetic testing. Left ventricular function was within normal ranges and not impaired. Interestingly, we found that LA size and secretory activity were increased in MFS patients, despite normal left ventricular filling pressures. This finding was even more pronounced in MFS patients with prior aortic surgery. A correlation between LA size or NT-proBNP levels and the type of pathogenic *FBN1* variant could not be identified. Right ventricular function and right atrial size were increased only in MFS patients that had undergone aortic surgery. In conclusion, these findings suggest that MFS leads to structural changes in the LA that are not solely resulting from left ventricular dysfunction, but probably can be considered a primary pathology of MFS.

## 1. Introduction

Marfan syndrome (MFS) is an autosomal-dominant multisystem connective tissue disorder with a prevalence of 1:5000 and a broad variability in phenotypic expression [1]. The cardiovascular, ocular and skeletal organ systems are frequently involved [2]. Other manifestations include the central nervous, integumental, and pulmonary systems [3]. Diagnosis is based on the revised Ghent criteria, with aortic root dilatation and lens luxation as the two cardinal manifestations of disease [4]. Life expectancy of MFS patients is mainly limited by cardiovascular complications, in particular the progressive dilatation of the proximal aorta with the risk of aortic dissection or fatal rupture [5].

A mutation in *FBN1*, encoding the extracellular matrix glycoprotein fibrillin 1, was identified as the cause of disease in the majority of MFS patients [6,7]. Fibrillin 1 is a main component of extracellular microfibrils and therefore plays a central role in the structural integrity of connective tissues [8]. In more recent studies, fibrillin 1-associated dysregulation of transforming growth factor (TGF)-β signaling has been described as an additional pathomechanism [9,10].

Apart from aortic root dilatation and its possible consequences, several studies in recent years reported on primary cardiomyopathy in MFS [3,11,12,13,14,15,16]. Evidence also exists for an involvement of the LA [13,17]. It has been shown in a mouse model that atria contain significantly more fibrillin 1 than ventricles [18]. Moreover, dysregulation in TGF-β signaling was associated with adverse remodeling of the atrial myocardium [19]. It therefore seems possible that impairment in LA function does not solely occur secondary to ventricular dysfunction, but could also be considered a primary pathology associated with MFS.

The aim of this study was to investigate cardiac function with a focus on the LA in a relatively large cohort of patients with MFS.

## 2. Materials and Methods

### 2.1. Study Design and Patient Selection

The local ethics committee approved this retrospective, single-center investigation (application EA2/120/16) following the principles of the Declaration of Helsinki. All 1165 patients who presented in our center for Marfan disease between 2016 and 2020 were retrospectively screened for eligibility. All patients were clinically assessed according to the revised Ghent nosology, and genetic test results were present in 94.8% of MFS patients [4]. Patients diagnosed with genetic aortic diseases other than MFS, such as Ehlers–Danlos syndrome or Loeys–Dietz syndrome, and patients with undefined diagnoses were excluded, as well as patients with known primary heart disease (ischemic cardiomyopathy (CMP) or CMP of any other etiology) and patients with more than mild mitral or aortic valve stenosis/regurgitation. A total of 231 adult patients with MFS and complete echocardiography and blood analysis were included for further analysis and divided into two subgroups—with (*n* = 85) and without (*n* = 122) prior aortic root surgery. According to that, patients who had had aortic surgery during the observation period (*n* = 24) were excluded for analysis, except for genotype–phenotype correlation where no differentiation between status of aortic surgery was made. In the same manner, by screening all cases who attended our specialized center in the given period, a control group was recruited from those who presented for suspected MFS or because of a close family member being diagnosed with MFS. They were included in our study if after clinical and in most cases genetic examination MFS or any other genetic aortic disease could be clearly ruled out.

### 2.2. Echocardiography—Technique and Measurements

Transthoracic echocardiography (TTE) was performed by one specialized physician using a state-of-the-art system (EPIQ 7, Philips Medical Systems DMC, Hamburg, Germany). Raw data was stored and analyzed using commercially available solutions (EchoPac, GE-Healthcare, Chicago, IL, USA). Standard echocardiographic images were recorded in parasternal short and long axes and apical two-, three-, and four-chamber views to evaluate left/right ventricular (LV/RV) and left/right atrial (LA/RA) dimensions and function utilizing established caliber and volumetric measurements. End diastolic and end systolic volume (EDV, ESV), fractional shortening (FS) and ejection fraction (EF) were retrieved by using the Teichholz formula. Valvular function was assessed using Doppler echocardiography. For many echocardiographic parameters, indices in relation to body surface area (BSA) were calculated. Aortic root measurements were taken from the parasternal long-axis view. Pulse-waved (PW) Doppler was used to assess LV diastolic function through quantification of transmitral inflow velocities during early (E) and late (A) diastole, deceleration time (DT) and isovolumetric relaxation time (IVRT). Moreover, septal and lateral mitral annular diastolic velocities (e’ septal/lateral) were measured by using tissue Doppler. E/e’ was calculated with an average e’ derived from septal and lateral e´ values.

### 2.3. Blood Sampling and Analysis

Blood samples were collected before conducting echocardiography in all analyzed cases. Serum concentrations of N-terminal pro-brain natriuretic peptide (NT-proBNP) were recorded.

### 2.4. Genetic Examination

Genetic testing for *FBN1* mutation was conducted after written informed consent during clinical standard procedure had been obtained from the affected individuals. Genetic analysis was performed using either *FBN1* sequencing by Sanger or an NGS-based gene panel as appropriate. Overall, 94.8% of MFS patients received a genetic test.

### 2.5. Statistical Analysis

Statistical analysis was performed with IBM SPSS Version 27 (SPSS Inc., Chicago, IL, USA).

If one study participant had more than one measurement of the same variable during the observation period, the mean was calculated and used for further analysis. All variables were checked for normal distribution, which was assumed when skewness was below |1|. One-way analysis of variance (ANOVA) was used for normally distributed parameters, and post hoc analyses were conducted using the Tukey test. For nonparametric analyses, the Kruskal–Wallis test and if needed Bonferroni post hoc tests were performed. Linear regression analysis was conducted and the Pearson correlation was used to calculate the strength of the association.

Results are shown as means ± standard deviation or medians ± interquartile range and are accepted as statistically significant when *p* < 0.05.

## 3. Results

### 3.1. Study Cohort

The study cohort characteristics are shown in Table 1. Out of 231 patients with MFS, 85 had undergone aortic surgery before the observation period and 122 had not. A total of 24 patients with MFS had aortic surgery during the intervention period and were therefore excluded for further analysis. Out of the 85 MFS patients who underwent surgery, 81 had replacement or repair of the aortic root/ascending aorta, and in 40% a composite graft was used. The group of MFS patients without aortic surgery consisted of more female patients and were on average younger in age. Body mass index (BMI) and body surface area (BSA) were similar between the groups (Table 1). As a control group, 106 healthy participants were included.

### 3.2. Left and Right Ventricular Function

MFS patients with prior aortic surgery presented with increased end systolic volume index (iESV) and lower LVEF compared to MFS patients without aortic surgery and control patients. Likewise, LV mass was higher in MFS patients and especially in MFS patients with aortic surgery in medical history. Analysis of diastolic function revealed a reduction in e’ and an increase in E/e’ for MFS patients with aortic surgery (Table 2). Of note, albeit with significant differences between the MFS and control group, absolute numbers of altered parameters were still within normal range. MFS with prior aortic surgery had significantly reduced TAPSE compared to the other two groups, as can be expected after cardiac surgery. Right ventricular end diastolic diameter was not different between the groups (Table 2).

### 3.3. Left and Right Atrial Size

MFS patients had significantly increased LA size compared to controls, as shown in Table 3. MFS patients with aortic surgery especially had an enlarged LA. Right atrial size for MFS patients with aortic surgery was increased, but still within normal range.

### 3.4. Increase in LA Size at Normal Filling Pressures

**Figure 1:**
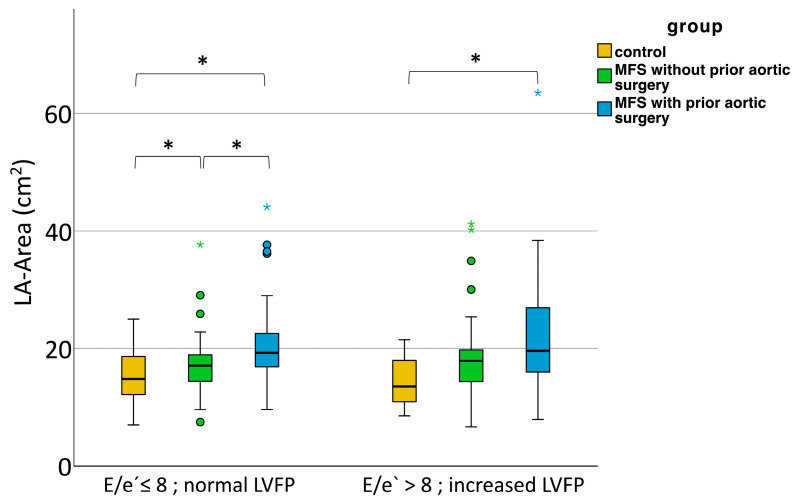
LA size in normal and elevated left ventricular filling pressures in healthy controls and patients with MFS with and without prior aortic surgery. Mild outliers that are more than 1.5 *IQR* below the first or above the third quartile (box) are represented by circles. Extreme outliers that are more than 3 *IQR* below the first or above the third quartile are represented by asterisks. LA = left atrium. LVFP = left ventricular filling pressure. MFS = Marfan syndrome. * *p* < 0.05 (Kruskal–Wallis and Bonferroni post hoc tests).

**Figure 2:**
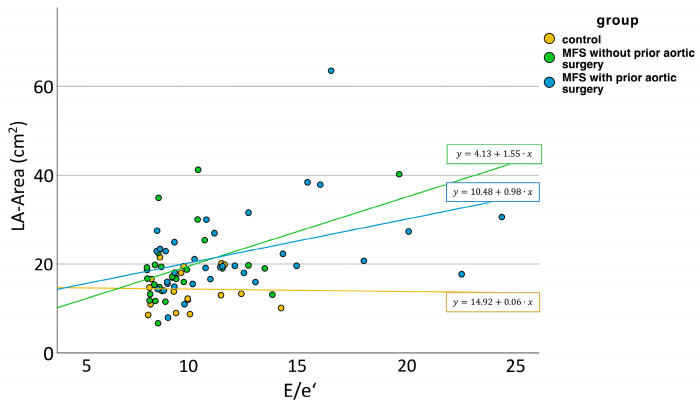
LA size in Relation to left ventricular filling pressure in healthy controls, patients with MFS with and without prior aortic surgery. LA = left atrium. MFS = Marfan syndrome.

To further elucidate the cause of LA enlargement in MFS patients, we examined the role of left ventricular filling pressures by using E/e’ as a surrogate marker. When E/e’ < 8, normal filling pressures can be assumed. Despite normal filling pressures, we could observe an increase in LA size for MFS patients without aortic surgery and even more for MFS patients with aortic surgery, indicating that LA enlargement is not solely due to increased filling pressures (Figure 1). Linear regression showed no correlation between LA size and E/e’. When observing LA size for E/e’ > 8, i.e., possible increase in filling pressures, we could also see an increase in LA size for MFS patients with aortic surgery (Figure 1). Here, linear regression between LA size and E/e‘ showed a correlation for MFS patients with aortic surgery (*p* = 0.018) and MFS patients without aortic surgery (*p* = 0.01), but not for controls (*p* = 0.093) (Figure 2).

### 3.5. Increase in NT-proBNP with Normal Filling Pressures

**Figure 3:**
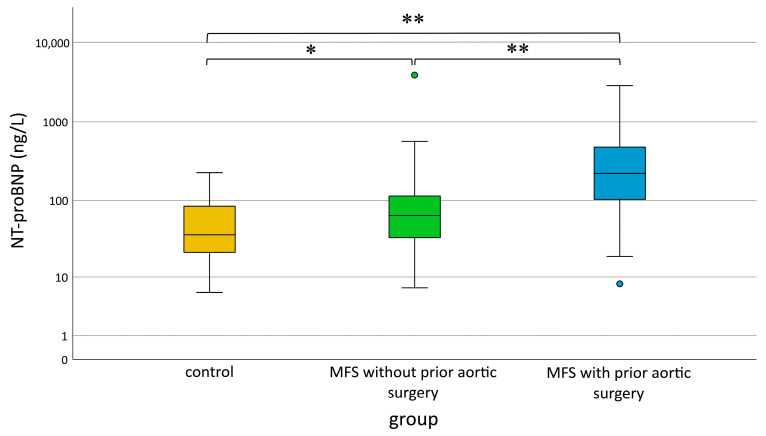
NT-proBNP in healthy controls (yellow), patients with MFS with (blue) and without (green) prior aortic surgery. Mild outliers that are more than 1.5 *IQR* below the first or above the third quartile (box) are represented by circles. MFS = Marfan syndrome, * *p* < 0.05, ** *p* < 0.001 (Kruskal–Wallis and Bonferroni post hoc tests).

To examine secretory activity of the LA, we measured serum NT-proBNP levels as a functional marker. We observed a significant increase in NT-proBNP serum levels for MFS patients with aortic surgery compared to the other two groups (Figure 3). Furthermore, MFS patients without aortic surgery also had significantly increased NT-proBNP levels compared to controls.

### 3.6. Influence of MFS Mutation on LA Size and Function

**Figure 4:**
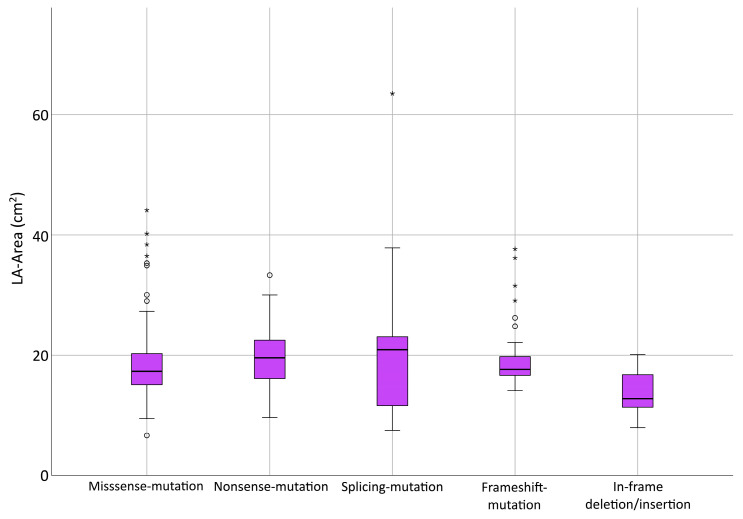
Genotype–phenotype relationship. Differences in LA size between types of pathogenic *FBN1* variant were not significant (Kruskal–Wallis test, *p* = 0.12). Mild outliers that are more than 1.5 *IQR* below the first or above the third quartile (box) are represented by circles. Extreme outliers that are more than 3 *IQR* below the first or above the third quartile are represented by asterisks. LA = left atrium.

In the next step, we examined the types of *FBN1* mutation, as shown in Table 4. Missense mutations were seen most frequently (46.3%). In search of genotype–phenotype correlations, we tested among those with a confirmed pathogenic *FBN1* variant (missense, nonsense, splicing, frameshift and in-frame deletion/insertion) differences in LA size or NT-proBNP levels (Figure 4). For both parameters, significant differences between type of mutation could not be found (*p* = 0.12 and *p* = 0.13).

## 4. Discussion

In this large cross-sectional study, we studied cardiac function with a specific emphasis on the LA in MFS patients with and without prior aortic surgery in comparison to a control group. We found that LA size and secretory activity is increased in MFS patients, especially in those with aortic surgery, despite normal left ventricular filling pressures. A correlation with the type of mutation could not be identified.

Systolic and diastolic ventricular function was within normal range for the majority of MFS patients. However, several studies reported on reduced left ventricular function in MFS patients in the absence of prior surgery, the so-called MFS cardiomyopathy [13,15]. It seems that newer imaging techniques, i.e., MRI and speckle-tracking echocardiography, are necessary to detect these subtle changes [20,21,22,23]. In this study using conventional echocardiography, we could see a decrease in LVEF and FS and increase in ESV only in MFS patients after aortic surgery. In MFS patients without prior surgical intervention, a slight tendency may be perceived. Of note, the values were still within normal ranges for all MFS patients. Probably, speckle-tracking technology would have helped to discriminate subtle changes in myocardial function. As described in the literature, none of our MFS patients had signs of overt heart failure. Right ventricular and right atrial parameters were impaired in operated MFS patients only, which can be expected after cardiac surgery [24].

Interestingly, even with conventional TTE techniques, we could detect a significant increase in LA size for MFS patients, which was more pronounced for those after aortic surgery. A strong association between LA enlargement and mortality has been demonstrated in a meta-analysis [25]. We excluded patients with significant mitral valvular disease, a factor that may influence atrial function, from our study. As LA function is directly dependent on left ventricular function, increased left ventricular filling pressure can lead to functional impairment and chronically to enlargement of the LA [26]. Few studies exist that focus on LA function in MFS patients. Abd El Rahman et al. detected reduced LA function in MFS patients by using speckle-tracking echocardiography and assumed that this might be caused by ventricular dysfunction [27]. In this current study, diastolic parameters were overall comparable between all three groups, with only E/e’ slightly elevated for MFS patients with prior aortic surgery. To further elucidate whether LA enlargement was secondary to enhanced left ventricular filling pressures, we examined LA size when normal filling pressures can be expected. E/e’ was used as a surrogate marker and an E/e’ < 8 considered indicative for normal filling pressures [28]. Our results show that LA size was significantly increased for MFS patients with and without surgery, even in the absence of increased filling pressures. For patients with possibly increased filling pressures, i.e., E/e’ > 8, we observed a further increase in LA size for MFS patients, plus a significant correlation between LA size and E/e’ values. Secretory activity, which we determined by measuring NT-proBNP levels, was significantly elevated in patients with MFS, even without prior aortic surgery. In patients who had undergone aortic surgery, levels of NT-proBNP were further increased. These findings implicate that MFS is associated with a left atrial pathology that is not a result of ventricular dysfunction, but most likely represents a primary structural disease. MFS patients with prior aortic surgery seem to be more susceptible, as they show larger LA than patients without surgery and additional LA enlargement at increased filling pressure. Explanations could be that (a) patients in need of an aortic surgery suffer from more severe disease and thus from more LA structural change, and/or (b) aortic surgery itself causes enough trauma that results in secondary cardiac pathologies.

On the molecular level, classic MFS is caused by mutations in fibrillin 1. Apart from being an important component of elastic microfibrils, fibrillin 1 stabilizes latent transforming growth factor β-binding proteins (LTBPs) in the extracellular matrix, which keeps transforming growth factor-β (TGF-β) in an inactive state. Therefore, malfunction of fibrillin 1 can result in excess TGF-β signaling, which turned out to be even more relevant for disease development than the disturbance in microfibril structure. Neptune et al. performed studies in fibrillin 1-deficient mice, an accepted model for MFS [29]. They showed that dysregulation of transforming growth factor-β (TGF-β) activation and signaling was causative for the development of disease-associated lung emphysema, which could be prevented by perinatal antagonism of TGF-β. In transgenic mice overexpressing myocardial TGF-β, overt levels of fibrosis were observed in the atria [19]. Since atria contain more fibrillin 1 than ventricles [18], a loss-of-function situation can probably lead to impaired atrial function when ventricular performance is still intact, as indicated by the results of this study.

The *FBN1* gene encoding fibrillin 1 is subject to a large number of mutations [30]. Moreover, the same mutation can lead to a variability of phenotypes. In this study, we could not find a correlation between LA size or NT-proBNP levels and the type of pathogenic variant, which confirms the current understanding that genotype–phenotype correlations in MFS are rather difficult [31].

This study has strengths and limitations. Its strengths include a large sample who were all clinically examined, assessed and diagnosed by the same experienced expert, which allows good comparability of measurements. Moreover, all patients underwent extensive diagnostics, including molecular genetic testing. The study is limited by its retrospective nature. Even though genetic aortic disease was ruled out for all subjects in the control group, certain pathologies could still exist, since they were referred to our center for clinical suspicion of connective tissue disease. However, it can be expected that using a completely healthy control group would probably even emphasize our results. E/e’ as a surrogate parameter can only be used for an estimation of filling pressures. The gold standard for an exact measurement would be to perform invasive catheterization, which (a) is not possible due to the type of study and (b) would have an unfavorable risk:benefit-ratio. NT-proBNP was considered a marker of atrial secretory activity [32,33], given that all patients were under stable disease and did not present symptoms of overt heart failure. However, measurement of ANP, MR-proANP or TGF-β would have been valuable additions. The potential impact of arterial hypertension on LA morphology could not be accounted for in this retrospective analysis, as there were no available data on blood pressure. LA area instead of volume was used for correlation analyses due to the higher number of available data, which is based on the fact that the LA was not the focus of examination and therefore volumes not measured in every patient. Taking the limitations into account, we consider this study hypothesis-generating. Further in-depth studies should follow.

## 5. Conclusions

In conclusion, patients with Marfan syndrome, especially those after aortic surgery, had significantly increased left atrial size in comparison to the control group. The results of this study indicate that MFS can lead to structural changes in the left atrium independently of filling pressures. A correlation between LA size and genotype could not be established.

## Figures and Tables

**Table 1:** Baseline characteristics.

	MFS without Aortic Surgery	MFS with Prior Aortic Surgery	Control
*n*	122	85	106
Female	74 (60.7%)	37 (43.5%)	52 (49.1%)
Age (years)	37.5 ± 15.0	47.4 ± 12.9	38.5 ± 14.3
Height (cm)	185 ± 10	188 ± 11	181 ± 11
Weight (kg)	75.5 ± 25.0	85.0 ± 29.0	69.5 ± 21.5
BMI (kg/m^2^)	22.1 ± 5.4	25.4 ± 6.5	21.2 ± 5.8
BSA (m^2^)	2.0 ± 0.3	2.1 ± 0.3	1.9 ± 0.2
HR (1/min)	69 ± 10	70 ± 12	75 ± 14
Medication			
ARB	82 (67.2%)	60 (70.6%)	21 (19.8%)
Losartan	73	41	16
Candesartan	7	7	4
Valsartan	2	12	1
Beta blocker	25 (20.7%)	60 (70.6%)	11 (10.4%)
ACE inhibitor	7 (5.7%)	14 (16.5%)	2 (1.9%)
Aortic diameter [mm]			
Anulus	23.6 ± 3.7	23.6 ± 4.2	22.0 ± 3.5
Bulbus	39.1 ± 7.0	34.3 ± 9.8	34.0 ± 6.4
ST-junction	30.8 ± 5.3	28.6 ± 6.5	28.3 ± 6.4
Ascending aorta	31.0 ± 5.5	29.6 ± 7.6	30.6 ± 5.7

Results are presented as means ± SD, medians ± IQR, or *n* (%) as appropriate. MFS = Marfan syndrome, BMI = body mass index, BSA = body surface area, HR = heart rate, ARB = angiotensin-receptor blocker, ACE = angiotensin-converting enzyme, ST = sinotubular.

**Table 2:** Ventricular function in MFS compared to controls.

	*n*	MFS without Aortic Surgery (1)	*n*	MFS with Prior Aortic Surgery (2)	*n*	Control (3)	*p* 1 vs. 2	*p* 1 vs. 3	*p* 2 vs. 3
Systolic function									
iLVIDd (mm/m^2^)	122	26.4 ± 4.2	85	24.7 ± 5.6	106	26.2 ± 3.8	ns	ns	ns
iLVIDs (mm/m^2^)	122	16.0 ± 2.7	85	16.7 ± 4.1	106	15.7 ± 2.6	ns	ns	ns
iEDV (mL/m^2^)	122	66.1 ± 18.6	85	63.6 ± 33.8	106	61.3 ± 17.5	ns	<0.05	ns
iESV (mL/m^2^)	122	19.9 ± 8.7	85	21.5 ± 14.7	106	17.8 ± 8.8	ns	ns	<0.001
FS (%)	122	39.8 ± 7.6	85	37.0 ± 10.2	106	40.1 ± 8.6	<0.01	ns	<0.01
EF (%)	122	69.7 ± 9.6	85	66.0 ± 13.6	106	70.6 ± 11.0	<0.01	ns	<0.001
Diastolic function									
E (cm/s)	121	75.0 ± 17.9	82	74.9 ± 25.0	103	74.7 ± 17.6	ns	ns	ns
A (cm/s)	121	55.4 ± 20.3	81	57.4 ± 22.4	103	52.4 ± 22.6	ns	ns	ns
E/A	121	1.3 ± 0.7	81	1.3 ± 0.6	103	1.4 ± 0.7	ns	ns	ns
e′ (cm/s)	122	10.7 ± 2.3	84	8.9 ± 2.2	98	11.8 ± 3.3	<0.001	<0.01	<0.001
IVRT (ms)	104	87.5 ± 25.8	64	82.8 ± 29.9	72	77.3 ± 29.0	ns	<0.001	<0.05
DT (ms)	121	176 ± 35	82	183 ± 53	102	170 ± 48	ns	ns	ns
E/e′	121	7.0 ± 2.0	82	7.7 ± 4.0	95	6.3 ± 3.0	<0.05	ns	<0.001
iIVSd (mm/m^2^)	121	5.2 ± 1.4	85	5.7 ± 1.5	106	4.8 ± 1.2	<0.05	ns	<0.001
iIVSs (mm/m^2^)	122	7.2 ± 1.7	85	8.0 ± 1.9	106	7.2 ± 1.7	<0.01	ns	<0.01
iPWd (mm/m^2^)	122	4.5 ± 0.7	85	4.7 ± 0.8	106	4.5 ± 0.8	ns	ns	ns
LV mass (g)	122	186.0 ± 87.1	85	234.0 ± 129.3	106	155.0 ± 69.3	<0.001	<0.001	<0.001
iRVIDd (mm/m^2^)	119	13.2 ± 2.6	79	13.4 ± 2.6	103	13.1 ± 2.5	ns	ns	ns
TAPSE (mm)	99	23.8 ± 6.7	65	17.6 ± 5.0	64	24.2 ± 6.7	<0.001	ns	<0.001

Data are presented as means ± SD or medians ± IQR as appropriate. For statistical testing, an ANOVA or Kruskal–Wallis test was performed. If significant, post hoc tests were added according to Tukey or Bonferroni, respectively. ns = non-significant. Parameters with ´i´ were indexed to body surface area. LVIDd = left ventricular diameter end diastolic, LVIDs = left ventricular diameter end systolic, EDV = end diastolic volume, ESV = end systolic volume, FS = fractional shortening, EF = ejection fraction, E = early diastolic mitral flow velocity, A = late diastolic flow velocity, e′ = early diastolic annular velocity (mean of septal and lateral), IVRT = isovolumetric relaxation time, DT = deceleration time of early diastolic flow velocity, IVSd = interventricular septum end diastolic, IVSs = interventricular septum end systolic, PWd = posterior wall thickness end diastolic, LV = left ventricle, RVIDd = right ventricular diameter end diastolic, TAPSE = tricuspid annular plane systolic excursion. MFS = Marfan syndrome.

**Table 3:** Atrial function.

	*n*	MFS without Aortic Surgery (1)	*n*	MFS with Prior Aortic Surgery (2)	*n*	Control (3)	*p* 1 vs. 2	*p* 1 vs. 3	*p* 2 vs. 3
LA area (cm^2^) +	111	17.1 ± 4.9	79	19.5 ± 8.2	93	14.4 ± 6.3	<0.001	0.003	<0.001
LA volume (mL) +	62	47.3 ± 28.4	47	57.4 ± 60.7	30	35.3 ± 21.1	0.12	0.05	<0.001
LAVi (mL/BSA) +	62	23.7 ± 16.9	47	27.0 ± 28.5	30	19.0 ± 10.3	0.42	0.07	0.002
RA area (cm^2^) +	76	13.8 ± 4.0	59	16.7 ± 7.6	47	13.8 ± 4.0	0.002	1	0.005

Data are presented as medians ± IQR. For statistical testing, a Kruskal–Wallis test and post hoc tests according to Bonferroni were calculated. LA = left atrium, LAVi = left atrial volume indexed to BSA, BSA = body surface area, RA = right atrium. MFS = Marfan syndrome.

**Table 4:** *FBN1* mutations in MFS.

Types of Mutation	Patients with MFS
*n*	231
Missense mutation	107 (46.3%)
Nonsense mutation	34 (14.7%)
Splicing mutation	19 (8.2%)
Frameshift mutation	31 (13.4%)
In-frame deletion/insertion	5 (2.2%)
Polymorphism	2 (0.9%)
Silent variants	8 (3.5%)
No mutation found	13 (5.6%)
Without genetic testing/missing data	12 (5.2%)

Results are presented as *n* (%). MFS = Marfan syndrome.

## Data Availability

The data presented in this study are available on request from the corresponding author. The data are not publicly available due to ethical restrictions.
